# The demographic history and adaptation of Canarian goat breeds to environmental conditions through the use of genome-wide SNP data

**DOI:** 10.1186/s12711-023-00869-0

**Published:** 2024-01-03

**Authors:** Gabriele Senczuk, Martina Macrì, Marika Di Civita, Salvatore Mastrangelo, Maria del Rosario Fresno, Juan Capote, Fabio Pilla, Juan Vicente Delgado, Marcel Amills, Amparo Martínez

**Affiliations:** 1 Department of Agricultural, Environmental and Food Sciences, University of Molise, 86100 Campobasso, Italy; 2 Animal Breeding Consulting S.L., 14014 Córdoba, Spain; 3 Universidad de Córdoba, 14071 Córdoba, Spain; 4 Department of Agricultural, Food and Forest Sciences, University of Palermo, 90128 Palermo, Italy; 5 Instituto Canario de Investigaciones Científicas, 38260 Tenerife, Spain; 6 CRAG, CSIC-IRTA-UAB-UB, Universitat Autònoma de Barcelona, 08193 Bellaterra, Spain

## Abstract

**Background:**

The presence of goats in the Canary Islands dates back to the late 1st millennium BC, which coincides with the colonization by the Amazigh settlers. However, the exact geographic origin of Canarian goats is uncertain since the Amazigh peoples were distributed over a wide spatial range. Nowadays, three Canarian breeds (Palmera, Majorera and Tinerfeña) are officially recognized, along with two distinct South and North Tinerfeña ecotypes, with the South Tinerfeña and Majorera goats thriving in arid and dry semi-desertic environments and the Palmera and North Tinerfeña goats are adapted to humid and temperate areas that are influenced by trade winds. Genotypes for 224 Canarian goats were generated using the Illumina Goat single nucleotide polymorphism (SNP)50 BeadChip. By merging these data with the genotypes from 1007 individuals of African and Southern European ancestry, our aim was to ascertain the geographic origin of the Canarian goats and identify genes associated with adaptation to diverse environmental conditions.

**Results:**

The diversity indices of the Canarian breeds align with most of those of the analyzed local breeds from Africa and Europe, except for the Palmera goats that showed lower levels of genetic variation. The Canarian breeds demonstrate a significant genetic differentiation compared to other populations, which indicates a history of prolonged geographic isolation. Moreover, the phylogenetic reconstruction indicated that the ancestry of the Canarian goats is fundamentally North African rather than West African. The ADMIXTURE and the TreeMix analyses showed no evidence of gene flow between Canarian goats and other continental breeds. The analysis of runs of homozygosity (ROH) identified 13 ROH islands while the window-based F_ST_ method detected 25 genomic regions under selection. Major signals of selection were found on *Capra hircus* (CHI) chromosomes 6, 7, and 10 using various comparisons and methods.

**Conclusions:**

This genome-wide analysis sheds new light on the evolutionary history of the four breeds that inhabit the Canary Islands. Our findings suggest a North African origin of the Canarian goats. In addition, within the genomic regions highlighted by the ROH and F_ST_ approaches, several genes related to body size and heat tolerance were identified.

**Supplementary Information:**

The online version contains supplementary material available at 10.1186/s12711-023-00869-0.

## Background

The origins of the Canarian goat breeds are not well known, but there is evidence that the Canary Islands were first colonized by the Amazigh settlers during the first century BC or later [1–3]. These settlers came mainly from West and Central North Africa [3], and they brought several domesticated animals, including sheep, pig and goats [4, 5]. Among these species, goats played a crucial role in their paleodiet [5]. Genetic analyses, using mitochondrial and microsatellite markers, revealed a common origin for the different Canarian breeds with high levels of haplotype sharing between goats from different Canarian islands [4, 6].

The Canarian natives were not familiar with navigation and remained isolated from the African continent until the fifteenth century [3]. Consistently, genetic analyses of Canarian goat populations have indicated a lack of haplotype sharing between Canarian and other gene pools (i.e. European and African) [7]. This is quite surprising especially when considering that during the Age of exploration, the Canary Islands became an important maritime cross-bridge that connected Europe with Africa and the Americas, thus facilitating the exchanges of livestock genetic resources [8]. If anything, at least a certain level of European matrilinear signature (mtDNA) harbored by Spanish goats brought to the Canary Islands during the last centuries should be expected. However, some genetic differentiation and a reduced gene flow between the Canarian and Spanish goats have also been outlined by microsatellite analyses confirming a secondary, if not very weak, role of the historical maritime exchanges from Spain in shaping the current genetic diversity of Canarian goats [9, 10]. In addition, the Canarian goats have contributed to the formation of several Creole American goat breeds [11].

Currently, the Canary Islands are home to more than 200,000 goats that are scattered throughout the archipelago representing a crucial asset in terms of milk and cheese production [12]. Since 2003, three Canarian goat breeds have been officially recognized: Palmera (from La Palma Island), Majorera (mainly widespread in Fuerteventura, Gran Canaria and Lanzarote) and Tinerfeña (Tenerife Island). Two Tinerfeña ecotypes that are adapted to dry (South Tinerfeña) and wet (North Tinerfeña) areas have been identified at the morphological [13] and genetic [6] levels. In addition, several morphotypes such as the feral Ajui-Costa and Esquinzo (Fuerteventura), Gran Canaria (Gran Canaria Island) and Gomera (Gomera Island) have been described [6]. Genetic analyses have suggested that these morphotypes should be considered as sub-populations of the Majorera breed [6].

A distinctive feature of the Canary Islands is the existence of highly differentiated climatic conditions across the different islands. For instance, Fuerteventura and Lanzarote have a dry subtropical climate with annual rainfall averages of 132 and 144 mm, respectively, and a semi desertic landscape with xerophyte vegetation [14]. This lack of rain is mostly due to these two islands being in close proximity to the African continent and to the absence of mountains (which favors the formation of clouds by raising and cooling the air). In contrast, La Palma Island has an average rainfall of 700–1000 mm and it is known as the “green island” because of its exuberant vegetation. This is attributed to the influence of trade winds which gather moisture, causing strong rainfall on the windward-facing slopes of the mountains [15]. Even within the islands, climatic conditions can vary dramatically. For instance, Southern Tenerife is dry and arid while Northern Tenerife has a higher rainfall and harbors Laurissilva woods again due to the influence of the trade winds [16]. This situation provides the opportunity to investigate the adaptation of Canarian goats, which likely come from a single ancestral gene pool [4], to highly divergent climatic conditions.

In this study, our aim was to investigate two fundamental questions. The first aim was to unravel the geographic origin of the Canarian goats. Previous studies based on mitochondrial and microsatellite markers [6, 7] did not conclusively address this question. In addition, the genetic analysis carried out by Colli et al. [17], which indicated a close affinity between the Canarian and West African goats, relied exclusively on the genotypes of a few Palmera individuals. Our second aim was to identify the genomic regions and genes, which might have been targeted by selection for environmental adaptation. This was achieved by comparing the gene pools of the Canarian breeds raised under humid conditions (Palmera and Northern Tinerfeña) with those adapted to dry conditions (Majorera and South Tinerfeña).

## Methods

### Sampling and genotyping

In total, 224 registered goats, including Palmera (n = 61), Majorera (n = 60), South Tinerfeña (n = 39) and North Tinerfeña (n = 64) were randomly collected from different flocks. Genomic DNA was extracted from whole blood samples using a standard phenol–chloroform method. Genomic DNA samples were genotyped with the Illumina Goat single nucleotide polymorphism (SNP)50 BeadChip (Illumina, San Diego, CA, USA). The raw genotype data from the Canarian goat breeds were merged with genotype data from 39 breeds (1007 individuals) from Europe and Africa that were retrieved from the Italian Goat Consortium 2 (IGC2) dataset, as described by Cortellari et al. [18]. The merged data were then mapped against the goat reference genome (ARS1.2 assembly). The IGC2 dataset included additional data for the Palmera breed (hereafter referred to as Palmera 2) that was previously genotyped by Manunza et al. [19]. The final dataset (hereafter referred to as the whole dataset), which included 45,149 SNPs and 1231 individuals, was obtained after applying the following SNP filtering criteria: a genotyping call rate of 90% (8,151 SNPs removed), a minor allele frequency (MAF) higher than 0.05 (23 SNPs removed) and a missing individual call rate of 10% (no individuals removed) using the software PLINK 1.9 [20]. Two additional subsets of data were built: the AFR-CAN dataset including only African and Canarian breeds (952 individuals) that was used for additional multidimensional scaling (MDS), neigbor-net and the TreeMix analyses and the CAN dataset including only the four Canarian breeds (224 individuals) that was used for all the analyses of signatures of selection (runs of homozygosity (ROH) islands and window-based F_ST_ analyses).

### Genetic diversity and population structure

Observed (Ho) and unbiased expected (uHe) heterozygosities (i.e. corrected for sample size) under Hardy–Weinberg equilibrium and the inbreeding coefficient i.e. F_IS_ = 1 − (mean(Ho)/mean(uHe)) were calculated with the R package dartR [21].

The MDS plot based on an identity-by-state (IBS) distance matrix was used to investigate the overall genetic relationship among the breeds by using the BITE R package [22]. This analysis was performed on both the whole dataset and the AFR-CAN dataset in order to have a reduced geographic scale while improving the resolution of genomic relationships. In addition, we used the maximum likelihood clustering approach implemented in the software ADMIXTURE [23] to infer the most likely number of genetic clusters (K) and the overall structure of the studied populations. The analysis was performed for K ranging from 1 to 30 using default parameters. The most predictive number of K was determined using a ten-fold cross-validation error procedure.

The genetic relationships between the studied populations were also explored for both the whole dataset and the AFR-CAN dataset, by calculating the Reynolds’ distance matrix using the R package *adegenet* [24]. These distances provide a measure of the genetic divergence by estimating the coancestry coefficient assuming that genetic drift is the main driver of the evolutionary differentiation. This matrix was then used to build a neigbor-net using an in-house script and then visualized using the SplitsTree program [25].

Finally, to infer a maximum-likelihood dendrogram and to test migration scenarios among the breeds, the TreeMix software [26] was used. For this analysis, only the subset AFR-CAN was used to assess the genetic relatedness and potential gene flow events between the Canarian and African breeds. The general analytical framework included three main steps. In the first step, a preliminary run was performed assuming migration edges from 0 to 15 using 10 replicates. In the second step, the optimal number of migration edges was determined using the linear method implemented in the *optm* function of the OptM R package [27]. In the final step, the best predicted number of migrations was used to build the consensus tree from 15 independent runs. This step included selecting the trees with the highest likelihood, removing duplicates and retaining the tree(s) with a unique topology. Steps (1) and (2) were performed using 1000 bootstraps and taking the linkage disequilibrium within blocks of 500 SNPs into account. The bezoar (*Capra aegagrus*), the ancestor of modern domestic goats, was used as an outgroup. The final consensus trees were visualized using the R package BITEV [22].

### Runs of homozygosity

Runs of homozygosity were detected using the PLINK 1.9 software [20], by setting the following criteria: a sliding window of 20 SNPs (-homozyg-window-snp 20); no genotyped heterozygous call (-homozyg-window-het 0); two missing genotypes allowed (-homozyg-window-missing 2); minimum number of SNPs within an ROH ≥ 20 (-homozyg-snp 20); a minimum ROH length of 2 Mb (-homozyg-kb 2000); a minimum SNP density of one SNP in 100 kb (-homozyg-density 100) and a maximum gap of 500 kb between consecutive homozygous SNPs (-homozyg-gap 500). According to these parameters, the F_ROH_ coefficient was calculated for each breed following the formula: F_ROH_ = L_ROH_/L_aut_, where L_ROH_ is the total length of ROH and L_aut_ is the length of the autosomal genome (2522 Mb).

### Selection scan based on ROH islands and FST analysis

Two analytical approaches were performed to identify genomic regions potentially under selection for environmental conditions: consecutive regions of homozygosity (ROH islands) and the F_ST_ window-based comparative analysis.

To identify the shared genomic regions that associated with ROH among individuals in each Canarian breed, the percentage of occurrences of SNPs in ROH was calculated by counting the number of times that a given SNP was in an ROH and by dividing this value by the number of animals in each breed, thus obtaining homozygosity estimates per locus. To identify ROH islands, the top 0.1% SNPs (99.9th percentile) of the percentile distribution were selected for each breed, separately. Adjacent SNPs above this threshold were merged into genomic regions.

In addition, the weighted F_ST_ statistics according to Weir and Cockerham [28] was used to detect regions under selection. Weighted F_ST_ were computed across windows of 500 kb in 250-kb steps using the VCFtools software v0.1.17 [29]. Windows that contained at least five SNPs within the top 0.05% F_ST_ (99.95th percentile) were considered to be significant. In addition, we used a multi-cohort approach in order to minimize the detection of possible false positive/negative outliers. The four Canarian breeds were first merged into two groups (meta-populations) based on their adaptation to wet (Palmera and North Tinerfeña) or dry (Majorera and South Tinerfeña) environments and then pairwise comparisons were made. Subsequently, four additional pairwise comparisons were tested by contrasting, each time, a breed adapted to wet with a breed adapted to dry environmental conditions.

All the significant candidate regions identified in this way were screened on the Genome Data Viewer (NCBI) using the ARS1.2 assembly to identify annotated genes.

## Results

### Genetic diversity and population structure

Diversity indices of the whole set of breeds showed an average of 0.380 for Ho and 0.391 for uHe. The maximum Ho value was recorded for the Malagueña breed (Spain) while the lowest for the Bezoar, however, among all the local breeds, the lowest Ho value was observed for the Palmera 2 breed (see Table 1). Concerning uHe, the maximum value was observed for the Mzabite breed (Algeria) and the lowest for the Palmera populations. In general, the Palmera populations (PAL, PAL_CA_ES and PAL2_CA_ES) showed the lowest diversity values while the other Canarian breeds (North and South Tinerfeña and Majorera) displayed values that were consistent with those of other Mediterranean and North African breeds. The majority of the breeds showed positive F_IS_ values including the four Canarian breeds (Table 1 and see Additional file 1: Fig. S1). The F_ROH_ coefficients for the Majorera and the South and North Tinerfeña breeds were in the range from 0.03 to 0.05, while for the two Palmera populations they reached values of 0.09 to 0.10 (Table 1 and see Additional file 2: Fig. S2).

**Table 1:** Names, geographic origin and codes for each goat breed and their sample sizes and genetic diversity indices

Breed name	Geographic origin	Breed code	n	Ho	Ho SD	uHe	uHe SD	F_IS_	F_ROH_
Canarian breeds									
Majorera	Canary	MAJ_CA_ES	60	0.380	0.139	0.385	0.118	0.013	0.030
Palmera 1	Canary	PAL_CA_ES	61	0.307	0.197	0.309	0.176	0.004	0.098
Palmera 2	Canary	PAL2_CA_ES	15	0.301	0.175	0.308	0.167	0.024	0.103
North Tinerfeña	Canary	TIN_CA_ES	64	0.363	0.142	0.364	0.134	0.002	0.052
South Tinerfeña	Canary	TIS_CA_ES	39	0.360	0.158	0.364	0.140	0.010	0.034
African breeds									
Barcha	Morocco	BAR_MA	4	0.403	0.256	0.422	0.165	0.044	0.046
Bezoar	Iran	BEZ_IR	7	0.275	0.199	0.359	0.176	0.235	0.170
Barki	Egypt	BRK_EG	134	0.410	0.100	0.421	0.093	0.026	0.036
Cameroon goat	Cameroon	CAM_CM	40	0.383	0.137	0.391	0.124	0.020	0.023
Djallonke	Burkina Faso	DJA_BF	12	0.363	0.191	0.368	0.154	0.014	0.023
Draa	Morocco	DRA_MA	4	0.403	0.255	0.420	0.165	0.039	0.061
Ghazalia	Morocco	GHA_MA	4	0.404	0.255	0.419	0.166	0.037	0.053
Guera	Mali	GUE_ML	25	0.392	0.171	0.374	0.141	-0.049	0.058
Maure	Mali	MAU_ML	14	0.398	0.172	0.393	0.133	-0.013	0.011
Moroccan goat	Morocco	MOR_MA	10	0.395	0.171	0.425	0.114	0.070	0.100
Mzabite	Algeria	MZB_AL	12	0.420	0.161	0.432	0.106	0.027	0.070
Naine	Mali	NAI_ML	17	0.362	0.177	0.364	0.151	0.006	0.031
Nubian	Egypt	NBN_EG	84	0.368	0.136	0.380	0.130	0.033	0.102
Noire de l’Atlas	Morocco	NDA_MA	4	0.395	0.255	0.420	0.167	0.058	0.129
Nord	Morocco	NOR_MA	4	0.405	0.253	0.430	0.157	0.059	0.064
Oasis	Egypt	OSS_EG	72	0.376	0.113	0.408	0.108	0.078	0.077
Peulh	Mali	PEU_ML	25	0.391	0.153	0.390	0.127	− 0.004	0.015
Red Sokoto	Nigeria	RSK_NG	21	0.376	0.150	0.397	0.124	0.051	0.069
Sahel	Burkina Faso	SAH_BF	15	0.383	0.169	0.392	0.132	0.023	0.035
Soudanaise	Mali	SDN_ML	24	0.387	0.151	0.389	0.127	0.006	0.011
Sahel	Nigeria	SHL_NG	21	0.395	0.148	0.402	0.119	0.017	0.017
Saidi	Egypt	SID_EG	60	0.392	0.113	0.415	0.102	0.056	0.061
Targui	Mali	TAR_ML	22	0.390	0.152	0.394	0.125	0.009	0.031
Tunisian	Tunisia	TUN_TN	23	0.416	0.135	0.422	0.101	0.015	0.031
West African Dwarf	Cameroon	WAD_CM	34	0.350	0.157	0.363	0.146	0.034	0.034
West African Dwarf	Nigeria	WAD_NG	21	0.354	0.167	0.366	0.147	0.031	0.036
European breeds									
Bermeja	Spain	BEY_ES	24	0.407	0.144	0.408	0.115	0.001	0.023
Bianca	Italy	BIA_IT	24	0.393	0.138	0.413	0.110	0.047	0.087
Garganica	Italy	GAR_IT	21	0.416	0.165	0.389	0.128	− 0.068	0.055
Garfagnina	Italy	GRF_IT	27	0.396	0.139	0.413	0.110	0.040	0.068
Mallorquina	Spain	MAL_ES	20	0.370	0.159	0.389	0.132	0.048	0.111
Messinese	Italy	MES_IT	24	0.417	0.136	0.420	0.104	0.006	0.013
Malagueña	Spain	MLG_ES	42	0.423	0.116	0.427	0.093	0.010	0.030
Maltese	Malta	MLT_MT	16	0.369	0.163	0.390	0.132	0.054	0.134
Murciano-Granadina	Spain	MUG_ES	20	0.411	0.148	0.412	0.113	0.003	0.034
Nera di Varzasca	Italy	NVE_IT	19	0.394	0.158	0.398	0.127	0.011	0.045
Blanca de Rasquera	Spain	RAS_ES	20	0.378	0.156	0.399	0.124	0.054	0.111
Vallesana	Italy	VLS_IT	22	0.347	0.166	0.369	0.145	0.061	0.150

Observed Heterozygosity (Ho) with standard deviation (SD), unbiased expected eterozygosity (uHe) with standard deviation (SD), inbreeding coefficient (F_IS_) and inbreeding coefficient based on ROH are reported

The first two dimensions of the MDS plot including the whole dataset accounted for 16.95% of the total variance, with the first dimension separating mainly the Mediterranean European breeds from the African breeds. The second dimension separated bezoars and Egyptian breeds from all the others (see Additional file 3: Fig. S3a). All the breeds from the Canary Islands clustered close to the breeds from West Africa (Mali, Nigeria and Cameroon). When considering the MDS plot of the AFR-CAN dataset, the Canarian breeds formed a separate cluster while all the African breeds were arranged along an axis that clearly outlines a geographic gradient from East to West (see Additional file 3: Fig. S3b).

**Fig. 1:**
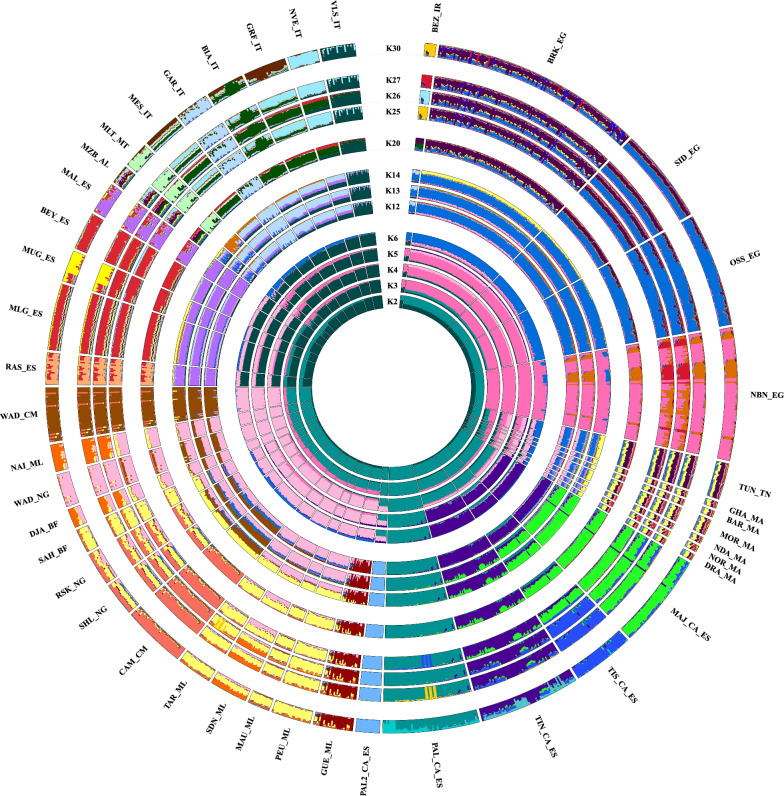
ADMIXTURE analysis with the main relevant K values of the 43 Southern European, African and Canarian goat breeds. For the full definition of breeds, see Table 1

The genetic structure analysis carried out with ADMIXTURE revealed that K = 20 is the most likely number of clusters. However, the genomic distribution of the main ancestral clusters can already be seen in the analyses comprising the first 5 K values (Fig. 1). At K = 2, the Italian and Spanish peninsular breeds are separated from all the other breeds, while K = 3 splits the bezoar and Egyptian breeds. Interestingly, the Canarian breeds showed an early separation from all the other populations at K = 4. At K = 5, a subsequent subdivision within the Canarian breeds, separated the Palmera from the Majorera, South Tinerfeña and North Tinerfeña breeds. In addition, at K = 13, the North Tinerfeña breed shows a distinctive identity, while the South Tinerfeña and Majorera breeds appear fairly similar in a single cluster until they subdivide at K = 26.

**Fig. 2:**
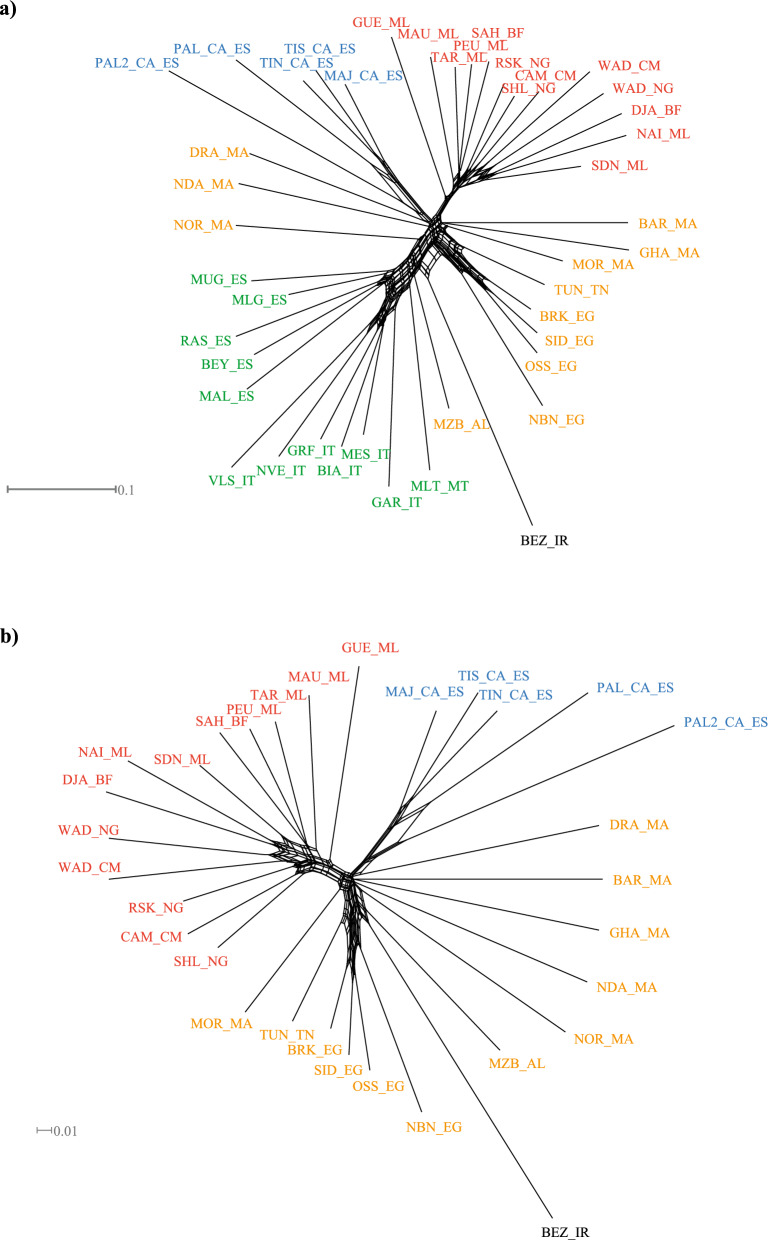
Neighbor-net based on Reynold’s distances of the whole dataset (Southern European, African and Canarian goat breeds) (**a**) and of the AFR_CAN dataset (only African and Canarian breeds). **b** The main clusters are colored according to geographic origin. The Canarian breeds are shown in blue, West African breeds in red, North African breeds in orange, European breeds in green and Bezoar in black. For the full definition of breeds, see Table 1

Neighbor-net graphs based on both the whole dataset (Fig. 2a) and the AFR-CAN dataset (Fig. 2b) revealed differentiated genetic clusters according to the geographic origin of the breeds. In both graphs, the Canarian breeds cluster nested within the African breeds although they appear more closely related to North Africa breeds especially those from Morocco.

**Fig. 3:**
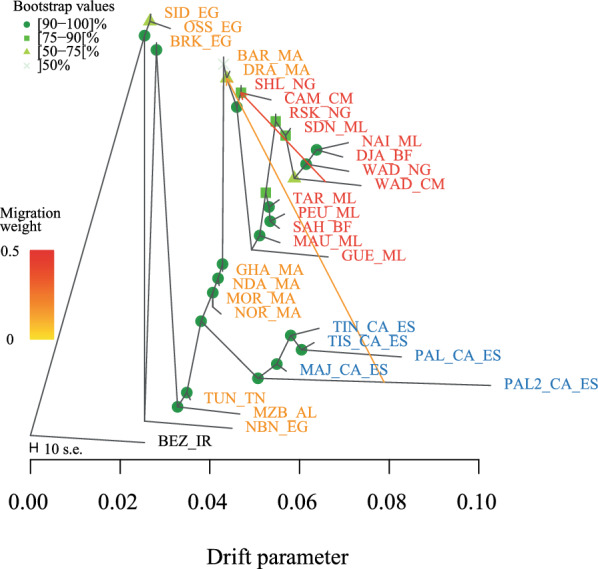
TreeMix analysis of the AFR_CAN dataset (only African and Canarian breeds) showing the best number of predicted migration edges. Bootstrap values are reported at each node while the strength of the migration weight is depicted by the intensity of the color of the arrows. The main clusters are colored according to geographic origin. The Canarian breeds are shown in blue, West African breeds in red, North African breeds in orange and Bezoar in black. For the full definition of breeds, see Table 1

The Treemix dendrogram obtained using the AFR-CAN dataset showed a robust topology with all nodes except three supported by bootstrap values higher than 75%. The consensus tree indicated a clear separation of the Canarian breeds, which are positioned close to the North African breeds especially to those from Morocco (Fig. 3). Concerning the genetic relationships among the Canarian breeds, the two Palmera populations (PAL_CA_ES and PAL2_CA_ES) do not cluster together with the Palmera 2 which is placed at a basal position while the Palmera breed is close to the South Tinerfeña breed. The linear method implemented in the *optm* function suggested two migration edges as the optimal number of migrations that best explain historical relationships. A first migration event is outlined from the West African Dwarf (WAD_CM form Cameroon) to the node including the Sahel and Cameroon goats (SHL_NG and CAM_CM, from Nigeria and Cameroon, respectively). Interestingly, a second migration edge is highlighted from the Palmera 2 population (PAL2_CA_ES, Canary) to the Moroccan Draa breed (DRA_MA).

### Selection scan based on ROH islands and FST analysis

**Fig. 4:**
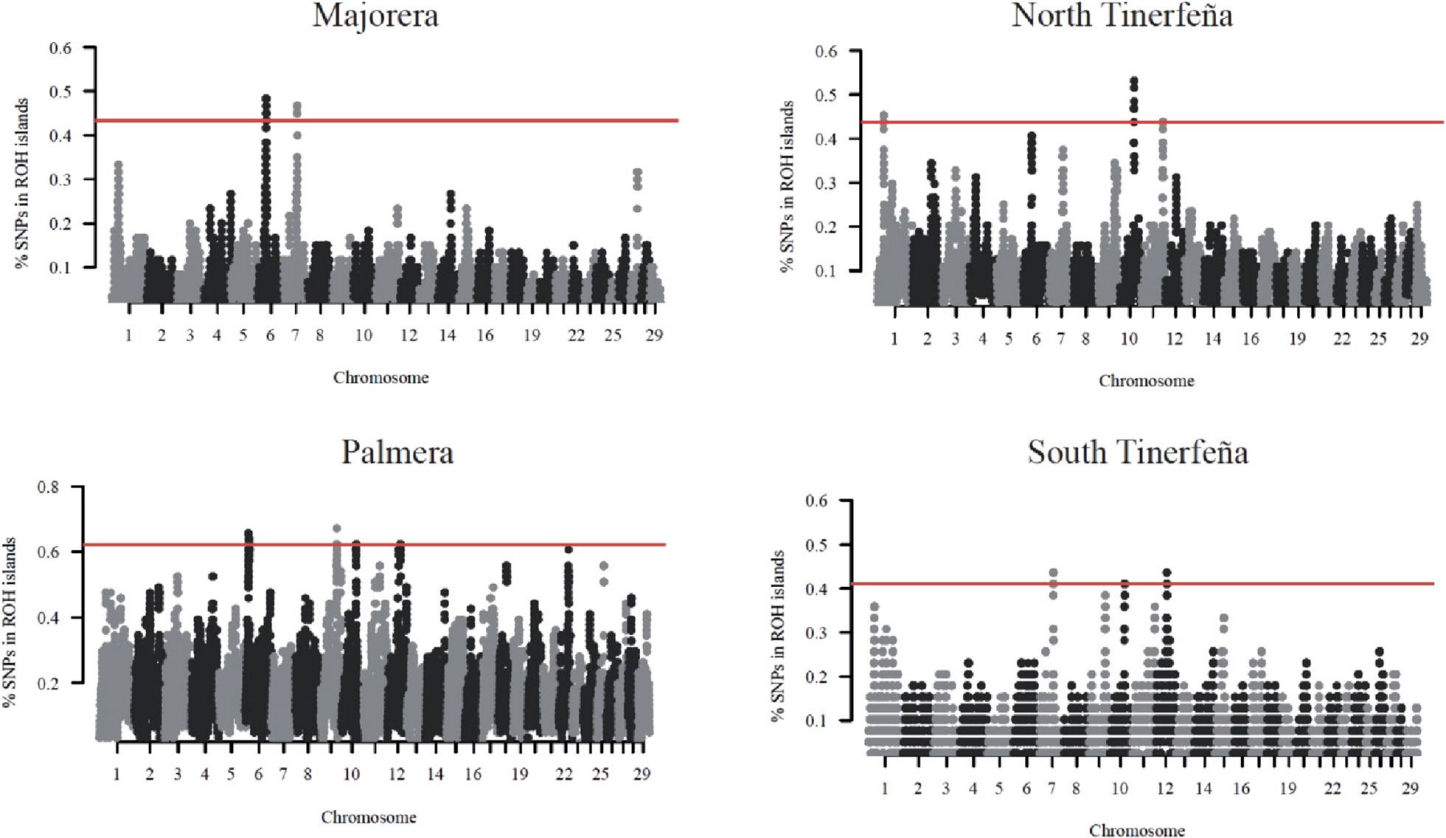
Manhattan plot of the ROH islands with the top 0.1% (99.9th percentile) shown above the red line

The ROH analysis carried out on the four Canarian breeds, identified 13 islands on seven *Capra hircus* (CHI) chromosomes (Table 2, Fig. 4). Within these genomic regions, 97 unique annotated genes were identified. The region located on CHI10 overlapped for three of the four Canarian breeds (North and South Tinerfeña and Palmera) and contained 18 annotated genes. Another ROH island located on CHI7 was identified in two breeds (MAJ and TIS) and encompassed 21 annotated genes. In addition, three ROH islands located on CHI06 were found in the Majorera and Palmera breeds, i.e. the 13.3–15.2 Mb; 16.4–18.1 Mb and 37.7–39.1 Mb regions that included nine, seven and five genes, respectively.

**Table 2:** List of significant ROH islands and corresponding genes identified in each breed

Breed	CHI	Start bp	End bp	N SNPs	Length Mb	Genes
MAJ	6	37,746,029	39,150,759	26	1.40	TRNASTOP-UCA, DCAF16*, NCAPG*, FAM184B*, LCORL*
	7	59,583,402	60,730,551	21	1.15	*CXXC5*, *UBE2D2*, *TMEM173*, *ECSCR*, *DNAJC18*, *SPATA24*, *PROB1*, *MZB1*, *SLC23A1*, *PAIP2*, *MATR3*, *SIL1*, *CTNNA1*, *LRRTM2*, *TRNAS-GGA*, *TRNAC-GCA*, *HSPA9*, *ETF1*, *EGR1*, *REEP2*, *KDM3B*
PAL	6	13,342,058	15,228,960	12	1.02	LARP7, ZGRF1, ALPK1*, TIFA*, AP1AR*, C6H4orf32*, NEUROG2, PITX2, ENPEP
	6	16,459,877	18,091,733	17	3.09	COL25A1, ETNPPL, OSTC, LEF1, HADH, SGMS2, PAPSS1*
	9	71,102,228	71,943,009	14	0.84	ADGB, STXBP5, SASH1
	10	66,619,056	67,062,439	10	0.46	*VPS18*, *RHOV*, *SPINT1*, *PPP1R14D*, *ZFYVE19*, *DNAJC17*, *C10H15orf62*, *GCHFR*, *RMDN3*, *RAD51*, *KNL1*, *TRNAS-GCU*, *RPUSD2, C10H15orf57, CHST14, BAHD1, IVD*, *KNSTRN*
	12	57,770,691	58,452,269	13	0.68	FRY, ZAR1L, BRCA2, N4BP2L1, N4BP2L2, PDS5B
TIN	1	27,667,084	28,183,186	12	0.51	GBE1
	10	66,062,165	67,331,443	24	1.29	*TYRO3, RPAP1, LTK, ITPKA, RTF1, TRNAW-CCA, NDUFAF1, NUSAP1, OIP5, CHP1, EXD1, INO80, CHAC1, DLL4, VPS18, RHOV, SPINT1, PPP1R14D, ZFYVE19, DNAJC17, C10H15orf62, GCHFR, RMDN3, RAD51, KNL1, TRNAS-GCU, RPUSD2, C10H15orf57, CHST14, BAHD1, IVD, KNSTRN, DISP2, C10H15orf52, INAFM2, PLCB2, ANKRD63, PAK6, BUB1B*, BMF
	11	95,578,638	96,126,324	10	0.52	PPP6C, RABEPK, HSPA5, GAPVD1, MAPKAP1, PBX3
TIS	7	59,583,402	60,848,376	21	1.27	*CXXC5*, *UBE2D2*, *TMEM173*, *ECSCR*, *DNAJC18*, *SPATA24*, *PROB1*, *MZB1*, *SLC23A1*, *PAIP2*, *MATR3*, *SIL1*, *CTNNA1*, *LRRTM2*, *TRNAS-GGA*, *TRNAC-GCA*, *HSPA9*, *ETF1*, *EGR1*, *REEP2*, *KDM3B*, FAM53C, CDC25C, GFRA3
	10	66,062,165	67,245,671	19	1.21	*TYRO3*, *RPAP1*, *LTK*, *ITPKA*, *RTF1*, *TRNAW-CCA*, *NDUFAF1*, *NUSAP1*, *OIP5*, *CHP1*, *EXD1*, *INO80*, *CHAC1*, *DLL4*, *VPS18*, *RHOV*, *SPINT1*, *PPP1R14D*, *ZFYVE19*, *DNAJC17*, *C10H15orf62*, *GCHFR*, *RMDN3*, *RAD51*, *KNL1*, *TRNAS-GCU*, *RPUSD2*, *C10H15orf57*, *CHST14*, *BAHD1*, *IVD*, *KNSTRN*, *DISP2*, *C10H15orf52*, *INAFM2*, *PLCB2*, *ANKRD63*, *PAK6*, *BUB1B*
	12	48,240,863	49,314,534	14	1.06	TRNAS-GGA

Chromosome (CHI), start and end position, number of SNPs (N), length of the ROH islands and relative genes are reported. All the genes detected in each significant ROH island are listed, with those that overlapped between goat breeds indicated in italics and those that overlapped between approaches (ROH and FST islands) indicated using the * symbol

**Fig. 5:**
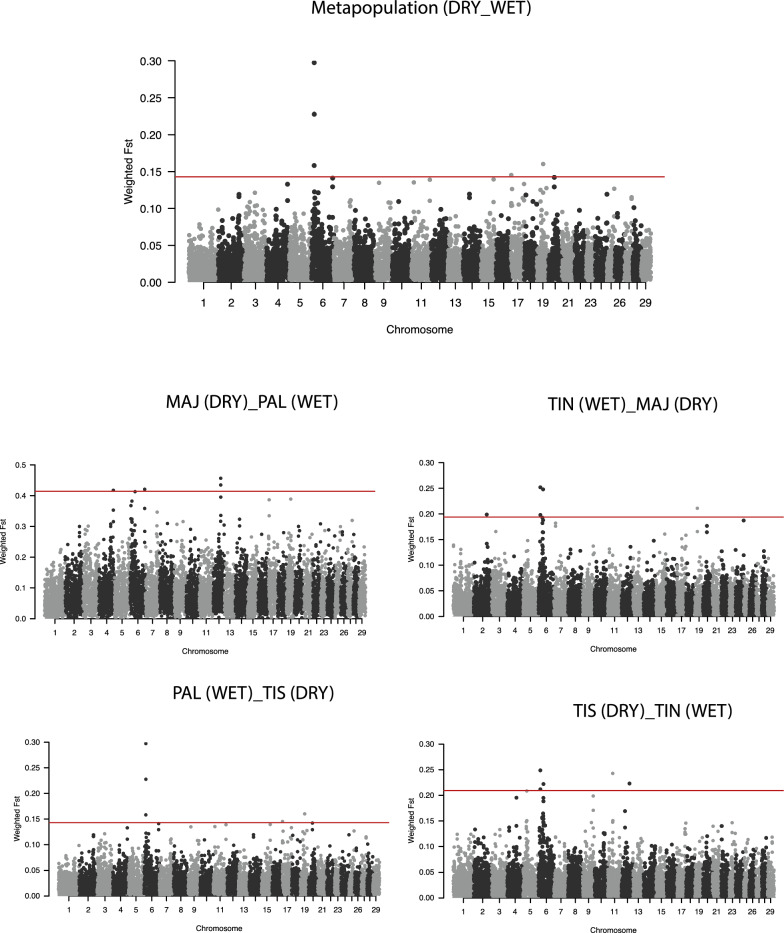
Manhattan plot of the weighted F_ST_ windows with the top 0.05% (99.95th percentile) shown above the red line. Metapopulation = MAJ + TIS vs PAL + TIN; DRY = dry condition; WET = wet condition

Concerning the Fst window-based approach, three genomic regions located on CHI6, 17 and 19, were identified in the metapopulation comparison i.e., wet (Palmera and North Tinerfeña) vs dry (Majorera and South Tinerfeña). In the single pairwise comparisons, 17 significant windows were detected (Table 3 and Fig. 5) that included a region located on CHI6 (13.75–14.25 Mb) that was significant in either the metapopulation or in three out of the four pairwise comparisons. However, because of slight differences in window size, only the significant window of the metapopulation comparison (13.5–14 Mb), which only partially overlapped with the aforementioned windows, included some genes (*ALPK1*, *TIFA*, *AP1AR*, and *C6H4orf32*). Interestingly, this region was also identified in the analysis of ROH islands for the PAL breed. Another region also located on CHI6 (37.75–38.25 Mb) was significant in two out of the four pairwise comparisons (PAL_MAJ and TIN_TIS) and in the analysis of ROH islands for the MAJ breed. This region includes five annotated genes (*FAM184B*, *DCAF16*, *NCAPG*, *LCORL* and *TRNAC-GCA*). Finally, another shared signal between the ROH and the F_ST_ window-based approaches was found on CHI6 (16–18 Mb), specifically in the PAL_TIS F_ST_ comparison and in the PAL ROH analysis. Due to discordances in window size, only one gene (*PAPSS1*) was consistently detected with the F_ST_ and ROH methods.

**Table 3:** List of significant windows and corresponding genes identified using the weighted F_ST_ approach

Comparison	CHI	Start (bp)	End (bp)	N SNPs	W Fst	Genes
Metapopulation	6	13,500,001	14,000,000	6	0.158	ALPK1*, TIFA*, AP1AR*, C6H4orf32*
	6	13,750,001	14,250,000	6	0.297	–
	6	14,000,001	14,500,000	9	0.227	–
	17	1,000,001	1,500,000	8	0.145	PISD, SFI1, EIF4ENIF1, DRG1, PATZ1, PIK3IP1, LIMK2, CRKL, RNF185, PLA2G3, INPP5J, SMTN, MORC2
	19	33,000,001	33,500,000	11	0.160	PIGL, NCOR1, TTC19, ZSWIM7, ADORA2B, AKAP10, ULK2
PAL_MAJ	4	110,750,001	111,250,000	12	0.417	PEX1, TRNAG-CCC, GATAD1, ANKIB1, KRIT1, LRRD1, AKAP9, TRNAC-GCA
	6	37,750,001	38,250,000	7	0.540	FAM184B*, DCAF16*, NCAPG*, LCORL*, *TRNAC-GCA*
	6	111,500,001	112,000,000	7	0.420	PROM1, TAPT1, LDB2
	12	57,750,001	58,250,000	9	0.456	FRY, ZAR1L, BRCA2, N4BP2L1, N4BP2L2, PDS5B
	12	5,800,0001	5,850,0000	8	0.434	–
MAJ_TIN	2	99,000,001	99,500,000	8	0.198	TANC1, WDSUB1, BAZ2B, TRNAC-ACA
	6	13,750,001	14,250,000	6	0.251	–
	6	14,000,001	14,500,000	9	0.197	–
	6	35,000,001	35,500,000	9	0.247	CCSER1, MMRN1, SNCA
	19	19,000,001	19,500,000	6	0.210	NOS2, LYRM9, NLK
PAL_TIS	6	13,750,001	14,250,000	6	0.407	–
	6	17,750,001	18,250,000	7	0.378	PAPSS1*
	6	111,500,001	112,000,000	7	0.404	PROM1, TAPT1, LDB2
	9	20,250,001	20,750,000	6	0.373	NUS1, GOPC, DCBLD1, ROS1, VGLL2
	21	62,250,001	62,750,000	12	0.367	–
TIN_TIS	6	13,750,001	14,250,000	6	0.248	–
	6	14,000,001	14,500,000	9	0.211	/
	6	37,750,001	38,250,000	7	0.222	FAM184B*, DCAF16*, NCAPG*, LCORL*, TRNAC-GCA
	11	38,000,001	38,500,000	6	0.242	EFEMP1, MIR217, MIR216B, CCDC85A
	12	60,500,001	61,000,000	5	0.223	NBEA, MAB21L1, TRNAE-UUC

Chromosome (CHI), start and end position, number of SNPs (N) within the windows, weighted FST with relative genes are reported. All the genes detected in each significant window using the weighted FST approach are listed, with those that overlapped between comparisons indicated in italics and those that overlapped between approaches (ROH islands and FST) indicated using the * symbol

## Discussion

### About the origin of the Canarian goat breeds

The arrival time of human and livestock on the Canary Islands is still debated, revealing a paradoxical timeline spanning almost 1000 years [30]. Indeed, several authors have suggested an early colonization during the 1st millennium BC, when the Amazigh peoples started occupying the Archipelago, while others argue for a more recent arrival dating back to the Roman period [1, 2, 31, 32]. The Imazighen ethnic and linguistic family extends through Morocco, Algeria, Tunisia, Libya, and Mauritania as well as small pockets of Mali, Niger, and Egypt. Indeed, Fregel et al. [3], analyzed the mitogenomes of ancient human remains from the seven main Canary Islands and showed that they mostly belong to Central North Africa, although some samples had a broader geographic origin range, including both West and Central North Africa, and, in some cases, Europe and the Near East. Our results contrast with those reported by Colli et al. [17], and show that most of the Canarian goats investigated in the current work have a closer affinity to North African than to West African breeds. These findings are consistent with the widely accepted hypothesis about the North African origin of the first Amazigh peoples who settled in the Canary Islands. The discrepant results between our study and that of Colli et al. [17] could be due to several reasons. From a technical point of view, it should be noted that although the MDS plot (see Additional file 3: Fig. S3) indicates an overlap between the Canarian breeds and those of West Africa, the Neighbor-net points out to a North African origin. In this regard, it should be noted that results from methods based on dimensionality reduction (i.e., MDS or PCA) should be considered with caution as recent studies have highlighted major issues concerning the replicability, accuracy and robustness associated with these techniques [33]. Indeed, we have noticed differences in the distribution of SNP distances when using the total dataset or the AFR-CAN subset (see Additional file 3: Fig. S3). This instability was also evident when the number of individuals of the Canary breeds was randomly reduced (data not shown). Conversely, phylogenetic reconstructions were consistent in showing a close genetic affinity between the Canarian and North African breeds. Indeed, both the Neighbor-net based on Reynolds’ distances and the TreeMix analysis showed a clustering following this scenario (Figs. 2 and 3). However, the inferred migration edges in the TreeMix showed an ancestral genetic exchange between the Palmera 2 and the base of the branch which mainly includes West African breeds, in accordance with what was previously reported [17]. This genetic connection might be related to past complex demographic dynamics characterized by successive colonizing waves settling in the Canary Islands over time as indicated by the analysis of mtDNA sequences from the Canarian indigenous populations that also included haplotypes from both West and Central North Africa [3]. However, our results do not provide clear evidence of more recent traces of introgression with West African breeds. In spite of the longstanding connection between the Iberian Peninsula and the Canary Islands, no evidence of admixture between Iberian and Canarian goats has been detected.

### Genome-wide relationships and variability of the Canarian goat breeds

Considering the overall genetic make-up within the archipelago, the genetic diversity indices indicated that all values except those of the Palmera breed, are in line with the majority of the other analyzed breeds. The low genetic variability in terms of uHe and Ho, together with the high values of inbreeding in the Palmera breed probably reflect the greater isolation of this population in accordance with the geographic setting of the archipelago [6, 19, 34]. In addition, studies relying on the analysis of mtDNA in Canarian goat populations have suggested a pre-Hispanic origin with a stepping-stone pattern of diffusion across the islands [4], therefore, a strong founder effect is the most likely cause of the reduced variability observed by us and others. Interestingly, population substructure was detected in the Palmera breed, with the Palmera 2 population displaying an ancestral admixed pattern while at higher K values this population was fairly homogeneous. In contrast, the Palmera 1 population displayed a homogeneous pattern at low K values while at higher K values it had a more admixed pattern. Such a controversial genetic make-up of the Palmera breed is also highlighted by the Neighbor-net graph and the TreeMix dendrogram (Figs. 2 and 3) in which the two populations do not cluster together. Unfortunately, the origin of the Palmera 2 sample is not known [17], however, our findings seem to indicate a mixed origin of this population followed by a bottleneck. The migration event outlined in the TreeMix analysis also support this interpretation, pointing to a Moroccan origin of the introgressed gene pool in the Palmera 2 population.

Concerning the other Canarian breeds, at high K values they clearly separated from each other, although a certain level of admixture can be observed. However, assessing whether this observed admixture is the result of recent gene flow or is caused by other processes such as retention of ancestral polymorphism is challenging especially when considering closely-related breeds. The observed admixture might also be associated with the topological inconsistency that we found in the TreeMix analyses. Indeed, in spite of the TreeMix scalability, evidence from a simulated dataset pointed out potential incorrect topologies especially in closely-related or admixed populations [35, 36].

### Signatures of adaptation to environmental conditions in the genomes of Canarian goats

In this study we compared native insular breeds adapted to dry (Majorera and South Tinerfeña) with those adapted to humid conditions (Palmera and North Tinerfeña) to identify regions under selection for environmental adaptation. The analytical approach, using the allele frequency-based inter-population genetic differentiation (F_ST_) and intra-population ROH, has been applied in several livestock species for the identification of genomic regions involved in phenotypic and environmental differences [37–40]. It has been widely corroborated that selection signature approaches based on different methods do not often provide overlapping results but can greatly improve the reduction of false positives and, in general, increase the robustness of the detected selection signals from other demographic processes [41]. Moreover, our analysis of signals of selection for divergent environmental conditions involved the comparison of Canarian populations that have a common ancestry [4], a feature expected to reduce false positive signals produced by genetic drift and other factors.

When considering the ROH approach, two genomic regions detected in more than one population were identified on CHI7 and 10. These two ROH islands encompass 64 annotated genes, some of which have already been shown to play a role in several traits related to adaptation and production. Interestingly, the same candidate region on CHI7 was detected in the breeds (Majorera and South Tinerfeña) that are more adapted to arid and dry conditions. At the same time, a number of studies that have focused on selective sweeps in African cattle adapted to tropical environments, with a special attention to regions associated with traits potentially related with adaptation to harsh conditions and thermotolerance have highlighted this very same region on chromosome 7 [42–46]. This region includes 21 genes, among which, several are involved in environmental thermal stresses responses (*ECSCR*, *DNAJC18*, *SLC23A1*, *HSPA9* and *SIL1*) and immunity (*TMEM173*, *MZB1* and *MATR3*). In particular, we found two heat shock protein genes (*DNAJC18* and *HSPA9*) and one gene encoding an oxidative stress response protein (*SLC23A1*) [44, 47]. These proteins play important roles in the response to heat stress by increasing the level of antioxidant enzymes that are important for the reduction of reactive oxygen species (ROS). In addition, all the breeds except the Majorera shared a candidate region on CHI10 that encompasses 18 genes present in the three breeds. However, none of these genes seem to have a known role in local adaptation to environmental variables.

The window-based F_ST_ approach identified several signals on CHI6 in more than one pairwise comparison. A convergent signal in this region was also found in ROH islands and among these candidate genes, two of them (*ALPK1* and *TIFA*) are directly involved in the innate immune response [48]. Another signal on CHI6 shared by the two approaches identified a region from about 37.7 to 39.1 Mb, which was detected in both the PAL-MAJ and TIN-TIS window-based F_ST_ comparisons as well as in the Majorera ROH analysis. To our knowledge, this region is of particular interest as it has also been detected as a candidate in a comparison between several other indigenous goats breeds from an arid hot environment in Egypt and European breeds that are raised under temperate environments [49]. This region encompasses five genes (*FAM184B*, *DCAF16*, *NCAPG*, *LCORL* and *TRNAC-GCA*), that have been suggested to be involved in body weight and stature in cattle, sheep and humans [50, 51]. In this context, it is well-known that body size is strongly related to warm climate responses and might impose severe constraints on adaptive variation [52, 53]. Moreover, this genomic region also overlapped with previously reported ROH islands in southern Italian goats [18, 54, 55] and in Merino and Merino-derived sheep breeds reared in different climatic zones [56]. One last genomic region shared between the two approaches was detected on CHI6 (16.4–18.2 Mb) in both the PAL-TIS window-based F_ST_ comparisons and in the Palmera ROH islands. However, because the two windows differ slightly in size, only one gene was found to overlap (*PAPSS1*). Although this gene does not seem to have a relevant function in adaptive traits, the downstream region highlighted in the ROH island, includes some interesting genes, such as *LEF1*, that has been demonstrated to promote the growth and development of hair follicles in Cashmere goats [57, 58]. Finally, it should be noted that the collagen gene on CHI6 (*COL25A1*) has been associated with adaptation to high altitudes in Ethiopian cattle [59].

## Conclusions

In summary, our results indicate that the Canarian goat breeds have a North African origin although an early West African contribution cannot be excluded. In addition, the absence of major admixture signals with either European or African breeds indicates that the Canarian goat populations presumably remained geographically isolated for a long period of time. In spite of such an isolation, all Canarian breeds except Palmera showed low levels of inbreeding, which suggests a relatively large founder population. This is not true for the Palmera breed, because due to its greater geographic isolation it may have suffered a strong genetic drift. Our study highlights the presence of several genomic regions which might be involved in adaptation to divergent environmental conditions. Interestingly, these regions contain several genes that are directly related to environmental thermal stress responses such as *DNAJC18*, *HSPA9* and *SLC23A1* while other genes (*FAM184B*, *DCAF16, NCAPG* and *LCORL*) are related to body size and growth, which are negatively correlated with thermal tolerance. Our results represent a first step to better understand the genes that could be central for local adaptation especially to harsh and arid conditions. We are confident that further genetic and phenotypic data will be essential to provide additional contributions to the understanding of the adaptive mechanisms, which are crucial for the ever-changing global environmental context.

### Supplementary Information

**Additional file 1: Figure S1**. Heterozygosities and F_IS_ by population. In each colored histogram, observed heterozygosity (Ho), expected unbiased (uHe) heterozygosity and the inbreeding coefficient (F_is_) are reported for each studied breed, respectively.

**Additional file 2: Figure S2.** Inbreeding F_ROH_ coefficients. Inbreeding molecular F_ROH_ coefficients of Southern European, African and Canarian goat breeds. The full definition of breeds is in Table 1.

**Additional file 3: Figure S3. **Multidimensional scaling plot (MDS). Description: Multidimensional scaling plot (MDS) of including (A) the whole dataset of Southern European, African and Canarian goat breeds and (B) the AFR-CAN dataset (see Table 1 for more details).

## Data Availability

The data that support the findings of this study are available on request from the corresponding author.
