# Supplementary material for: The demographic history and adaptation of Canarian goat breeds to environmental conditions through the use of genome-wide SNP data
Source: Genet Sel Evol. 2024 Jan 3;56:2. doi: 10.1186/s12711-023-00869-0 (PMC10763158; doi:10.1186/s12711-023-00869-0)

Figure S1. In each colored histogram, observed heterozygosity (Ho), expected unbiased (uHe) heterozygosity and the inbreeding coefficient (Fis) are respectively reported for each studied breed.

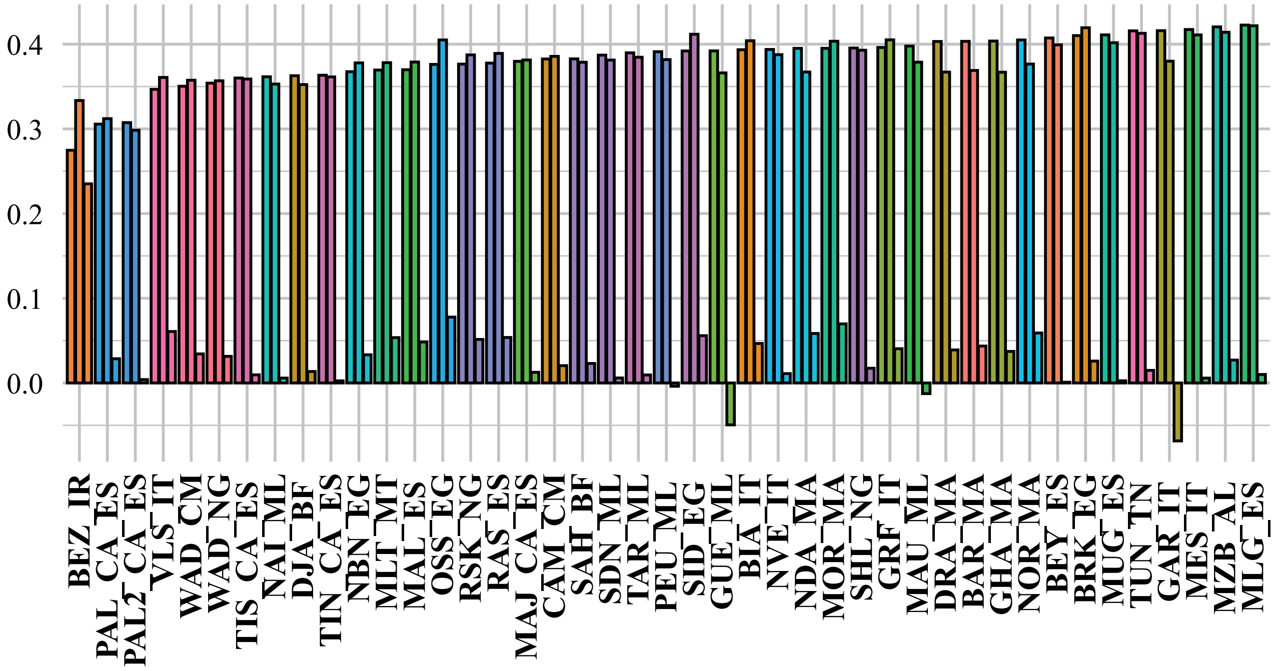

Supplement: Supplementary file 1 — Additional file 1: Figure S1. Heterozygosities and FIS by population. In each colored histogram, observed heterozygosity (Ho), expected unbiased (uHe) heterozygosity and the inbreeding coefficient (Fis) are reported for each studied breed, respectively. [file 12711_2023_869_MOESM1_ESM.pdf]
