# Supplementary figures and images for: The demographic history and adaptation of Canarian goat breeds to environmental conditions through the use of genome-wide SNP data
Source: Genet Sel Evol. 2024 Jan 3;56:2. doi: 10.1186/s12711-023-00869-0 (PMC10763158; doi:10.1186/s12711-023-00869-0)

**Figure S2.** Inbreeding coefficients calculated using ROH (FROH). for each breed.

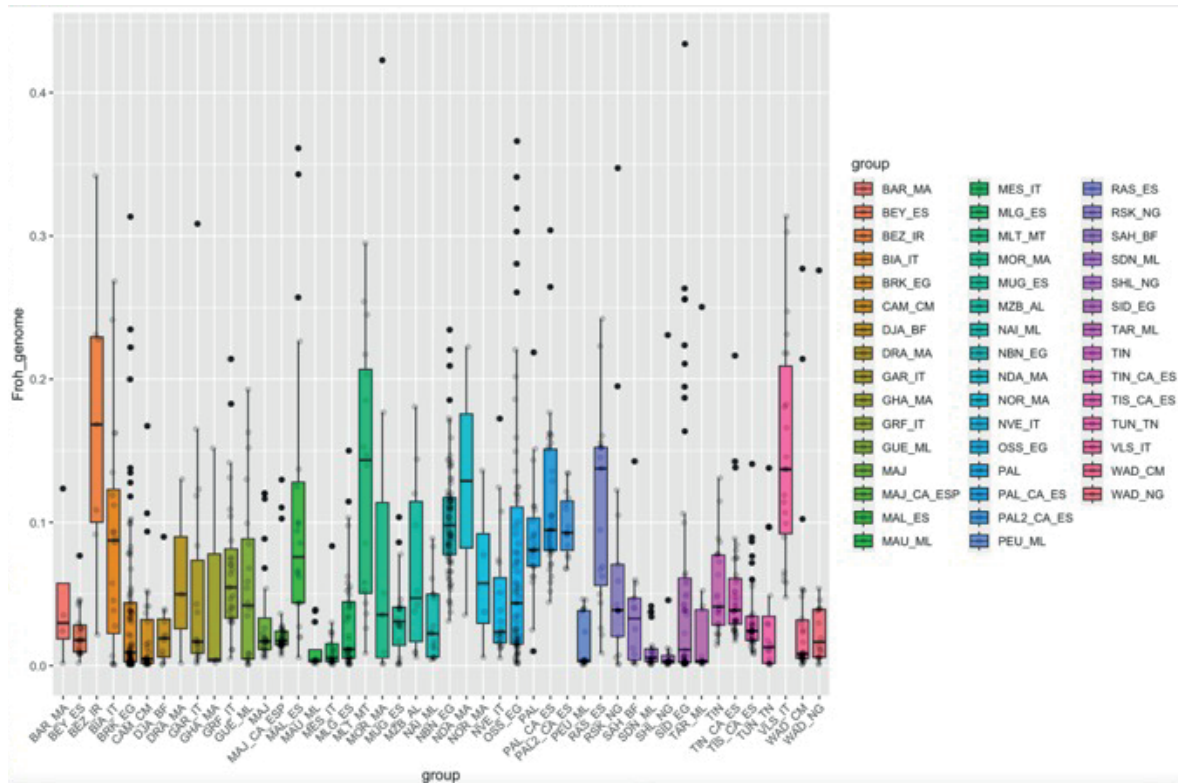

Supplement: Supplementary file 2 — Additional file 2: Figure S2. Inbreeding FROH coefficients. Inbreeding molecular FROH coefficients of Southern European, African and Canarian goat breeds. The full definition of breeds is in Table 1. [file 12711_2023_869_MOESM2_ESM.pdf]
