# Supplementary material for: The demographic history and adaptation of Canarian goat breeds to environmental conditions through the use of genome-wide SNP data
Source: Genet Sel Evol. 2024 Jan 3;56:2. doi: 10.1186/s12711-023-00869-0 (PMC10763158; doi:10.1186/s12711-023-00869-0)

Figure S3. The Multidimensional Scaling Plot (MDS) of the analyzed breeds using the whole dataset a) and the AFR-CAN dataset b). See Table 1 for more details.

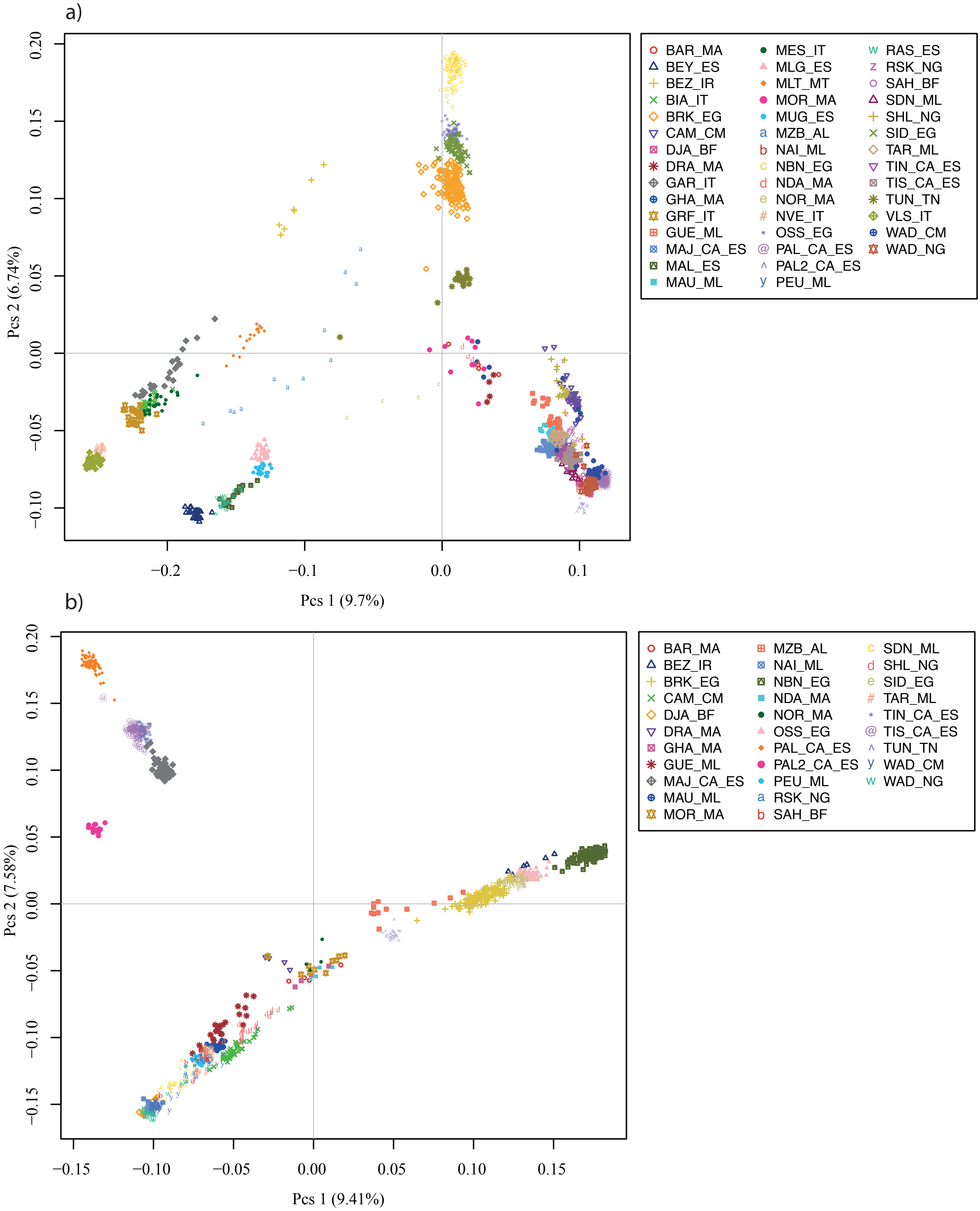

Supplement: Supplementary file 3 — Additional file 3: Figure S3. Multidimensional scaling plot (MDS). Description: Multidimensional scaling plot (MDS) of including (A) the whole dataset of Southern European, African and Canarian goat breeds and (B) the AFR-CAN dataset (see Table 1 for more details). [file 12711_2023_869_MOESM3_ESM.pdf]
